# Freehand suturing to apply traction during endoscopic submucosal dissection

**DOI:** 10.1055/a-2282-9928

**Published:** 2024-04-03

**Authors:** Jochen Weigt, Cosima Göring, Verena Keitel

**Affiliations:** 1 Department of Gastroenterology, Hepatology and Infectious Diseases, Otto-von-Guericke University Magdeburg, Magdeburg, Germany

The technique of freehand suturing in gastrointestinal endoscopy has evolved through technical developments such as knotless barbed wire sutures and needle manipulators, with initial publications showing promising results 1 2 . The main advantage of freehand suturing over currently used device-based suturing is the ease of the technique. The method of freehand suturing offers a variety of possible applications, but these are not yet well established. Besides closing of mucosal or complete wall defects of the gastrointestinal tract, one of our first thoughts was the use of the suture string to apply tension to the mucosa while performing resections such as endoscopic submucosal dissection (ESD) or submucosal tunnel preparation. We present the technique of freehand suture traction during ESD in a porcine stomach.

First, electrocautery marks were applied to the mucosal surface to indicate the lesion for resection. Using a HybridKnife (ERBE Elektromedizin, Tübingen, Germany), we gained access to the submucosal space and injected dyed saline solution. After preparing two entry points to the submucosal space at the proximal and distal part of the lesion, we sutured the proximal part of the lesion using a barbed wire (V-Loc 180, 1/2 circle taper end needle 3–0; Covidien, Dublin, Ireland) ( Fig. 1 , Fig. 2 , Video 1 ). Steering of the needle was performed using a distal cap attachment (MTW Endoskopie, Wesel, Germany) and a needle holder (SutuArt; Olympus, Hamburg, Germany). This barbed wire was then sutured to the opposite side of the gastric lumen, and carefully tensed until the lesion mucosa was lifted in a tent-like fashion, thus opening the submucosal space for further preparation ( Fig. 3 ). Preparation was performed using the HybridKnife in preciseSECT mode. Each mucosal plane was cut after adjacent submucosa had been prepared, and the expanding resection flap was peeled away using endoCUT mode. If the suture string lost tension, the barbed wire was carefully tensed again by gently pulling the suture through the suture tract, thus guaranteeing tenting and traction of the submucosal layer. As our intention was to perform a peel away preparation with maximal widening of the preparation plane, we tried to tether the mucosal specimen flap away from the oral resection margin by suturing it to a more distal part of the opposite gastric wall.

**Fig. 1:**
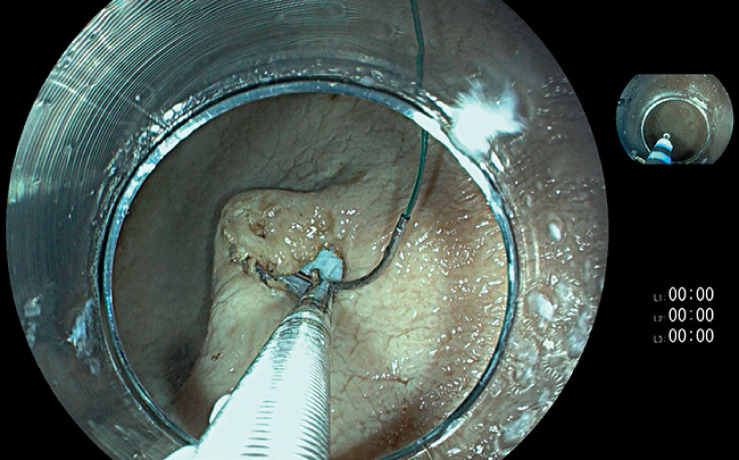
Initial needle passage from the prepared submucosal entry point into the specimen.

**Fig. 2:**
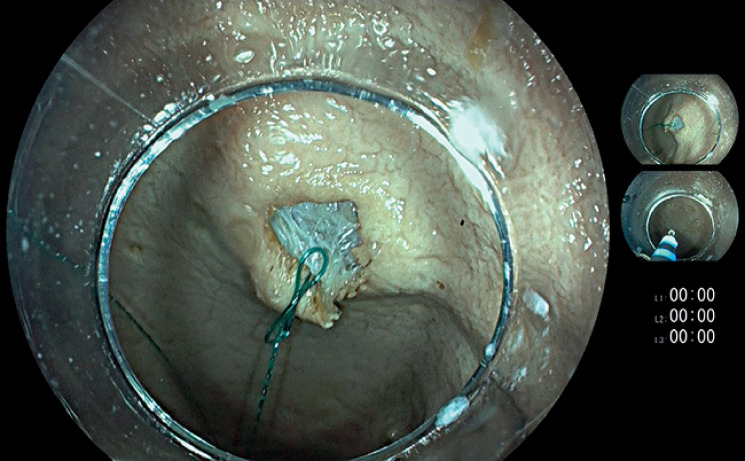
Fixation of the suture to the specimen.

**Fig. 3:**
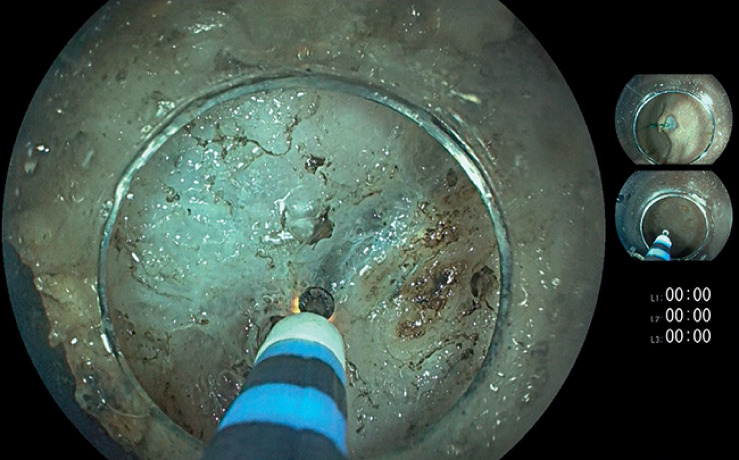
Opening of the resection plane during submucosal preparation.

Suture tethering of a mucosal flap using freehand suturing technique during endoscopic submucosal dissection.Video 1

The rationale for starting the procedure by preparing two submucosal entries points was to enable access to the distal end of the resection specimen after tethering. During the procedure, the suture slipped out of the mucosal anchoring site, but was easily reattached with two stitches at opposite sides.

This is the first report of using barbed wire freehand suturing to apply traction during ESD. This technique is easy to perform and has low costs. The advantages of this method over other traction techniques, such as rubber band clipping, is that the amount of tension can easily be adapted while the intervention is ongoing by simply pulling the tethering suture slightly through the mucosal surface, as it will not slip backward due to the barbed wire structure allowing only unidirectional movement of the suture. Another clear advantage is that the specimen is easy to grasp and retrieve from the body after completing the resection and that it is clearly marked by the suture. This may help to orientate the specimen for better histological workup and orientation of resection margins.

Endoscopy_UCTN_Code_TTT_1AO_2AG_3AD

## Notes

Endoscopy E-Videos https://eref.thieme.de/e-videos
*E-Videos* is an open access online section of the journal *Endoscopy* , reporting on interesting cases and new techniques in gastroenterological endoscopy. All papers include a high-quality video and are published with a Creative Commons CC-BY license. Endoscopy E-Videos qualify for HINARI discounts and waivers and eligibility is automatically checked during the submission process. We grant 100% waivers to articles whose corresponding authors are based in Group A countries and 50% waivers to those who are based in Group B countries as classified by Research4Life (see: https://www.research4life.org/access/eligibility/ ). This section has its own submission website at https://mc.manuscriptcentral.com/e-videos .
